# Tumour endothelial cells in high metastatic tumours promote metastasis via epigenetic dysregulation of biglycan

**DOI:** 10.1038/srep28039

**Published:** 2016-06-13

**Authors:** Nako Maishi, Yusuke Ohba, Kosuke Akiyama, Noritaka Ohga, Jun-ichi Hamada, Hiroko Nagao-Kitamoto, Mohammad Towfik Alam, Kazuyuki Yamamoto, Taisuke Kawamoto, Nobuo Inoue, Akinobu Taketomi, Masanobu Shindoh, Yasuhiro Hida, Kyoko Hida

**Affiliations:** 1 Vascular Biology, Frontier Research Unit, Institute for Genetic Medicine, Hokkaido University, Sapporo 060-0815, Japan; 2 Department of Vascular Biology, Hokkaido University Graduate School of Dental Medicine, Sapporo 060-8586, Japan; 3 Department of Cell Physiology, Hokkaido University Graduate School of Medicine, Sapporo 060-8638, Japan; 4 Division of Stem Cell Biology, Institute for Genetic Medicine, Hokkaido University, Sapporo 060-0815, Japan; 5 Department of Gerodontology, Hokkaido University Graduate School of Dental Medicine, Sapporo 060-8586, Japan; 6 Department of Gastroenterological Surgery I, Hokkaido University Graduate School of Medicine, Sapporo 060-8638, Japan; 7 Department of Oral Pathology and Biology, Hokkaido University Graduate School of Dental Medicine, Sapporo 060-8586, Japan; 8 Department of Cardiovascular and Thoracic Surgery, Hokkaido University Graduate School of Medicine, Sapporo 060-8638, Japan

## Abstract

Tumour blood vessels are gateways for distant metastasis. Recent studies have revealed that tumour endothelial cells (TECs) demonstrate distinct phenotypes from their normal counterparts. We have demonstrated that features of TECs are different depending on tumour malignancy, suggesting that TECs communicate with surrounding tumour cells. However, the contribution of TECs to metastasis has not been elucidated. Here, we show that TECs actively promote tumour metastasis through a bidirectional interaction between tumour cells and TECs. Co-implantation of TECs isolated from highly metastatic tumours accelerated lung metastases of low metastatic tumours. Biglycan, a small leucine-rich repeat proteoglycan secreted from TECs, activated tumour cell migration via nuclear factor-κB and extracellular signal–regulated kinase 1/2. Biglycan expression was upregulated by DNA demethylation in TECs. Collectively, our results demonstrate that TECs are altered in their microenvironment and, in turn, instigate tumour cells to metastasize, which is a novel mechanism for tumour metastasis.

Tumour metastasis causes the high mortality rates that are associated with cancer. During the first stage of the metastatic process, tumour cells migrate through a vascular wall (intravasation) and then travel to target organs^1^,^2^. Tumour blood vessels provide a route for distant metastasis^3^. Indeed, highly vascularized tumours exhibit high metastatic potential^4^,^5^. The morphologies and functions of tumour vasculatures are known to differ from those of their normal counterparts^6^,^7^. Recent studies, including ours, revealed that tumour endothelial cells (TECs), components of tumour blood vessels, also differ from normal endothelial cells (NECs) in various aspects, including their angiogenic properties^8^, gene expression profiles^9^ and responses to growth factors^10^,^11^ and chemotherapeutic drugs^12^,^13^,^14^. Furthermore, TECs are cytogenetically abnormal^15^,^16^. We recently demonstrated the heterogeneity of TECs using two different types of these cells: HM-TECs from highly metastatic melanomas [HM-tumour, A375-SM (super-metastatic)] and LM-TECs from low metastatic melanomas (LM-tumour, A375). HM-TECs exhibited greater pro-angiogenic activities than LM-TECs did, which was concomitant with the upregulation of angiogenesis-related genes^14^. These results indicated that TECs acquired specific features in response to their surrounding environment.

Here, we investigated the roles of TECs in tumour metastasis by utilizing the two aforementioned different tumour models (HM-tumours and LM-tumours) and the corresponding TECs (HM-TECs and LM-TECs) isolated from these tumours. Our results provide clear evidence that TECs actively promote tumour metastasis, particularly during intravasation, through the secretion of the small leucine-rich proteoglycan, biglycan. In addition, we found that biglycan expression was upregulated by DNA demethylation of its promoter region in TECs. Collectively, to the best of our knowledge, these results demonstrate for the first time a novel mechanism for tumour metastasis.

## Results

### HM-TECs promote tumour cell intravasation and metastases

LM-tumour and HM-tumour cells were subcutaneously xenografted into nude mice. The two melanoma cell lines were derived from identical human tumours but with significantly different metastatic potentials; A375 cells barely metastasize, whereas A375SM cells (generated from A375 cells by repeatedly re-inoculating metastasized tumour cells) develop lung metastases^17^. Consistent with previous reports^17^, more mice with HM-tumours than with LM-tumours developed lung metastases (Supplementary Fig. S1A) and tumour cells were detected in intra-blood vessel areas of HM-tumours (Supplementary Fig. S1B), which also demonstrated more angiogenic properties (Supplementary Fig. S1C).

**Figure 1:**
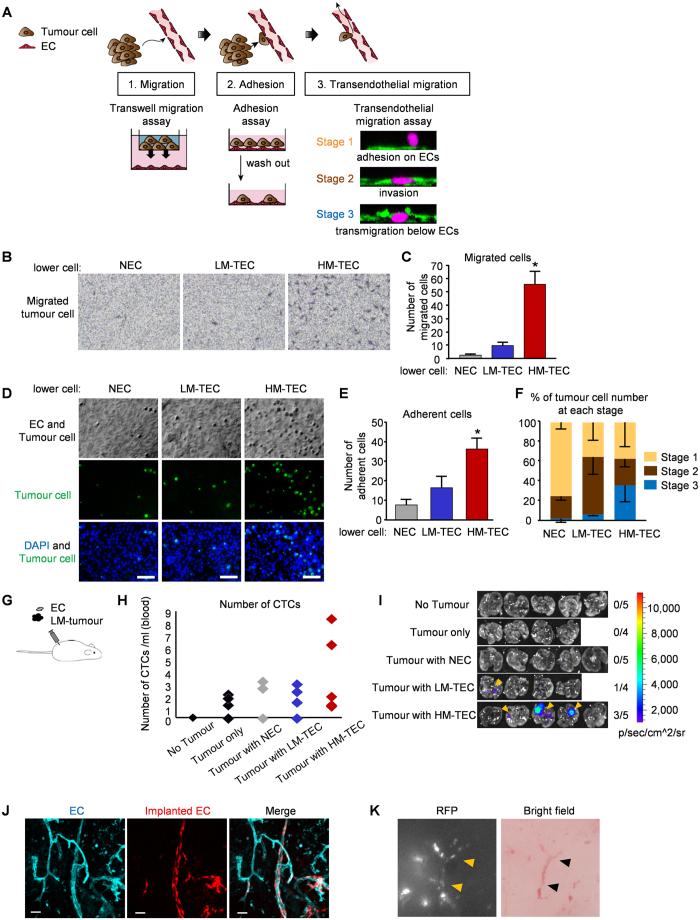
HM-TECs promote tumour cell intravasation and metastasis. (**A**) Schematic of the steps involved during tumour intravasation: migration, adhesion and transendothelial migration. (**B,C**) LM-tumour cells that migrated to the underside of the membrane were photographed (**B**) and counted (**C**). (*P < 0.01 versus LM-TECs and NECs, one-way ANOVA. Data are mean ± SD, n = 6 fields). (**D**) Representative photomicrographs of bright-field (upper panels) and fluorescence (middle panels) microscopic images of adherent tumour cells on EC monolayers after co-culture for 30 min. Merged images of adherent tumour cells (green) and DAPI (blue) are also shown in lower panels. Scale bar = 100 μm. (**E**) Adherent tumour cells with a FITC-anti-human HLA antibody were counted (*P < 0.01 versus LM-TECs and NECs, one-way ANOVA. Data are mean ± SD, n = 6 fields). (**F**) Tumour cells at each stage were counted and plotted as a percentage of total cells (Data are mean ± SD, n = 3 independent experiments). (**G**) Schematic illustration of experimental methods. LM-tumour cells were co-xenografted with one type of EC (HM-TECs, LM-TECs, or NECs) into nude mice (n = 4 or 5). (**H**) Circulating RFP-positive tumour cell numbers were determined by flow cytometry (n = 4 or 5). (**I**) Tumour cell luminescence intensity in the lungs (arrowhead) was detected using IVIS Spectrum. (**J**) All blood vessels in tumours were visualized by Alexa Fluor 647-GS-1B4 lectin (cyan). Specimens were observed under a fluorescence microscope. Of note, implanted TECs (red) were connected to host ECs. Arrowhead indicates co-localization. Scale bar = 20 μm. (**K**) Tumour vessels were imaged using a fluorescence stereomicroscope. A fluorescence image (left) and a bright-field image (right) show that the vasculatures comprising implanted ECs (expressing RFP) and containing red blood cells. Arrowheads indicate co-localization.

In hematogenous metastasis, tumour cells detach from the primary site and enter the blood vasculature. This process of intravasation can be divided into three steps: 1) tumour cell migration toward endothelial cells (ECs), i.e., “migration”; 2) arrest on ECs, i.e., “adhesion”; and 3) migration through the endothelium, i.e., “transendothelial migration”^18^ (Fig. 1A). We investigated the involvement of TECs in these steps *in vitro*. TECs were isolated from HM- and LM-tumours^14^,^19^ and NECs were isolated from the dermis of tumour-free nude mice (Supplementary Fig. S1D). The characteristics of these cells were confirmed as ECs through the expression of EC markers and the absence of human tumour cell contamination with the lack of hHB-EGF expression (Supplementary Fig. S1E–S1G). Tumour cells migrated more efficiently toward HM-TECs than toward other ECs (Fig. 1B,C and Supplementary Fig. S2A,B), which suggested that a soluble factor(s) secreted by HM-TEC had attracted tumour cells. Next, the adhesiveness of LM-tumour cells to monolayers of each type of EC was compared. Tumour cells more efficiently adhered to a HM-TEC monolayer than they did to monolayers of the other ECs (Fig. 1D,E). To further analyse the groove under the EC monolayer of LM-tumour cells (an *in vitro* model of intravasation), a transendothelial migration assay^20^,^21^ was performed, in which the positional relationship between EC monolayers and tumour cells was classified into three different stages (Fig. 1A). On NEC or LM-TEC monolayers, most tumour cells were observed to be in Stage 1 or 2. In contrast, on HM-TEC monolayers, 40% of tumour cells were in Stage 3, which demonstrated that tumour transmigration was enhanced on the HM-TEC monolayer (Fig. 1F).

To evaluate the contribution of each EC to transendothelial migration and subsequent intravasation and metastasis, LM-tumour cells and ECs were subcutaneously co-implanted into nude mice (Fig. 1G and Supplementary Fig. S2C). Circulating tumour cells (CTCs) in peripheral blood were evaluated by flow cytometry. The highest number of RFP-expressing CTCs was observed in mice bearing tumours co-implanted with HM-TECs (Fig. 1H). There were no significant differences among the groups, but these results suggested that HM-TECs instigate LM-tumour cell metastasis by inducing intravasation. Next, Luciferase-LM-tumour cells and tdtomato-ECs (Supplementary Fig. S2D) were co-xenografted and lung metastases were evaluated by IVIS Spectrum. Lung metastasis, which could not be observed when LM-tumour cells alone were subcutaneously inoculated (Fig. 1I), was significantly increased from that with LM-TECs (1/4) or NECs (0/5) when a tumour was co-implanted with HM-TECs (3/5) (Fig. 1I). However, the tumour sizes were comparable among these groups (Supplementary Fig. S2E). The mean MVD of LM-tumours co-implanted with HM-TECs was the highest among all tumour groups (Supplementary Fig. S2F). In *in vivo* primary tumours, the red fluorescence signals originating from co-implanted ECs were detected in lectin-positive blood vessels to some extent (Fig. 1J) and the vasculature comprising these ECs contained red blood cells (Fig. 1K), which suggested that implanted ECs had participated in the formation of functional blood vessels in cooperation with the host’s vasculature.

### HM-TECs express high levels of biglycan via demethylation of its promoter region

**Figure 2:**
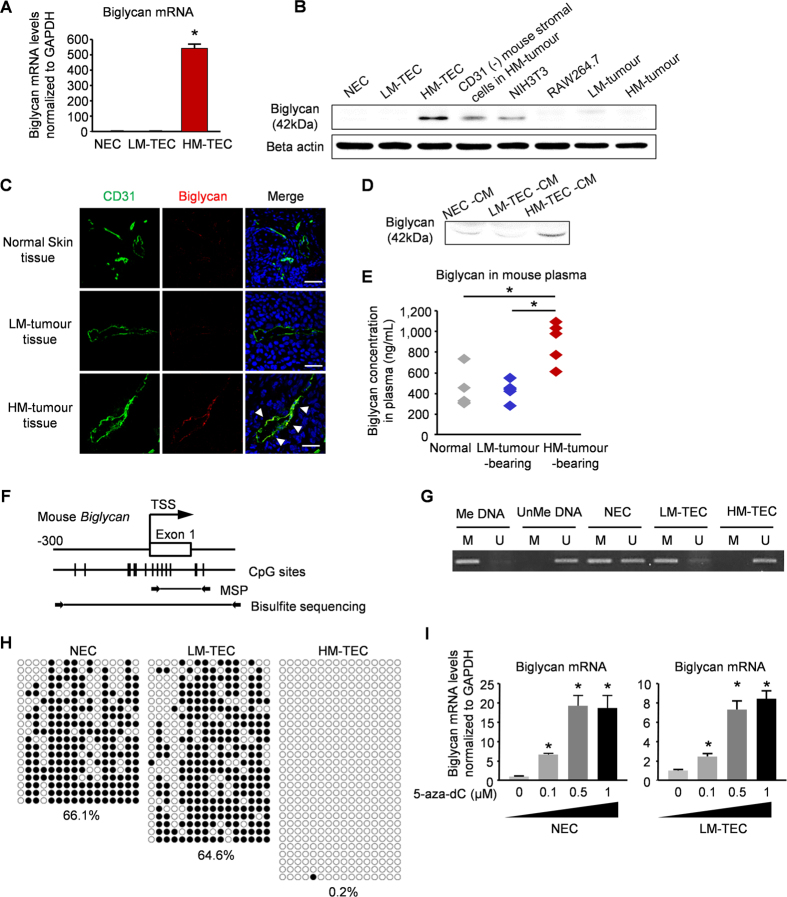
HM-TECs express and secrete biglycan via demethylation of its promoter. (**A**) Biglycan expression was evaluated by real-time PCR (*P < 0.01 versus LM-TECs and NECs, one-way ANOVA. Data are mean ± SD, n = 4 real-time RT-PCR runs). (**B**) Biglycan protein levels in various cells were analysed by western blotting. (**C**) Biglycan expression in tumours dissected from mouse and normal dermal tissues. Arrowhead indicates CD31 and biglycan co-localization. Scale bar = 50 μm. (**D**) Biglycan protein in conditioned medium (CM) from each type of EC was analysed by western blotting. (**E**) Plasma biglycan levels were determined by ELISA for each mouse group (*P < 0.01 versus Normal and LM-tumour-bearing, one-way ANOVA. Data are mean ± SD, n = 5). (**F**) A schematic diagram of the CpG sites in the mouse biglycan promoter; vertical ticks indicate CpG sites; arrowheads indicate the specific primers used for MSP and bisulfite sequencing analyses. (**G**) A representative image of the MSP analysis of the biglycan promoter. Me DNA, methylated control DNA; UnMe DNA, unmethylated control DNA; M, methylated PCR product; U, unmethylated PCR product. (**H**) Bisulfite sequencing analysis of the biglycan promoter in ECs. The white and black circles indicate unmethylated and methylated CpG dinucleotides, respectively. The results are from at least 19 individually sequenced clones. Quantification of DNA methylation is shown. (**I**) Relative biglycan mRNA levels in NECs and LM-TECs treated with 5-aza-dC at the indicated doses [*P < 0.01 versus 5-aza-dC (0 μM), one-way ANOVA. Data are represented as mean ± SD, n = 4 real-time RT-PCR runs].

By comparing the gene expression profiles of TECs and NECs, we previously identified biglycan among the upregulated genes, which is a secreted protein of small leucine-rich proteoglycans (SLRPs)^22^. We also found that biglycan secreted from TECs induces their pro-angiogenic phenotype in an autocrine manner, such as cell migration^23^. Biglycan expression was upregulated in HM-TECs relative to that in other ECs or other cell types, including HM- or LM-tumour cells (Fig. 2A,B). Furthermore, strong biglycan expression was observed in HM-tumour vessels, but was hardly observed in LM-tumour vessels or in normal skin vessels (Fig. 2C). In addition, biglycan was only detected in HM-TEC conditioned medium (CM) (Fig. 2D and Supplementary Fig. S3A). The biglycan levels in the plasma of HM-tumour-bearing mice were higher than those in the LM-tumour-bearing mice or non-tumour mice (Fig. 2E). These results suggest that HM-TECs secrete biglycan and attract tumour cells in a paracrine manner.

Based on the differences between HM-TECs and LM-TECs in the tumour microenvironment, we speculated that HM-TECs acquired their specific characteristics from tumour-derived factors. Thus, we cultured LM-TECs in CM derived from HM-tumour cells, LM-tumour cells, or LM-TECs (Control) for 2 days, after which biglycan mRNA expression was evaluated. Biglycan mRNA in LM-TECs was upregulated after treatment with HM-tumour-CM (Supplementary Fig. S3B) and the number of migrating LM-tumour cells increased when HM-tumour CM-treated LM-TECs were plated in the lower chambers of transwells (Supplementary Fig. S3C). These results suggest that LM-TECs acquire a “HM-TEC-like” phenotype under the influence of HM-tumour-derived factors. Biglycan upregulation in HM-TECs was maintained for several months, although all ECs had been cultured under the same conditions without the addition of any tumour-derived factors. We speculated that this difference may be attributable to a difference in DNA methylation. The transcription start site (TSS) in the biglycan promoter was identified using MethPrimer software^24^, and the DNA methylation status of the area around the TSS was examined by methylation-specific polymerase chain reaction (PCR; MSP) and a bisulfite sequencing analysis (Fig. 2F). MSP data revealed that the biglycan promoter in HM-TECs had markedly less methylation than that in other ECs (Fig. 2G). Consistently, bisulfite sequencing showed that the biglycan promoter was significantly demethylated in HM-TECs (Fig. 2H). Biglycan expression was indeed increased when LM-TECs and NECs were treated with the demethylating agent 5-aza-dC (Fig. 2I). These data indicate that epigenetic modification causes biglycan upregulation in TECs.

### HM-TEC-derived biglycan induces tumour cell intravasation and metastasis

**Figure 3:**
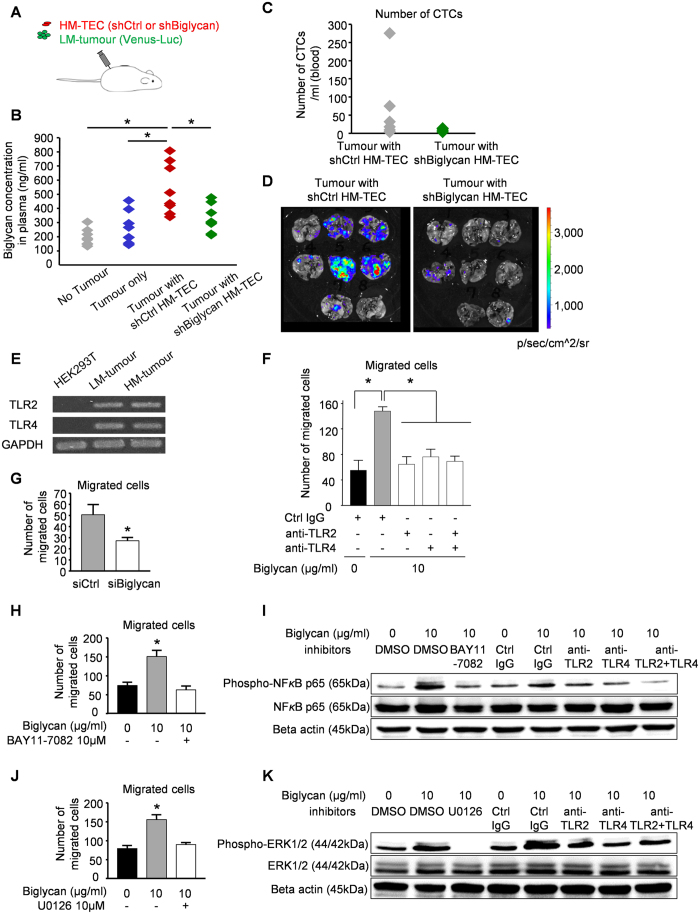
HM-TEC-derived biglycan induces tumour cell intravasation and metastasis through the activation of NF-κB and ERK Signalling via TLR2 and TLR4. (**A**) LM-tumour cells were subcutaneously implanted along with HM-TECs transfected with shBiglycan or those transfected with control shRNA (shCtrl); n = 8. (**B**) Plasma biglycan levels were determined by ELISA for each mouse group (*P < 0.01 versus No Tumour, Tumour only and Tumour with shBiglycan HM-TEC, one-way ANOVA, n = 8). (**C**) The number of Venus-positive circulating tumour cells was analysed by flow cytometry (n = 8). See also Supplementary Fig. S3G. (**D**) Tumour cell luminescence intensity in the lungs was detected using IVIS Spectrum. (**E**) TLR2 and TLR4 mRNA expression levels in HM- and LM-tumour cells were determined by RT-PCR. (**F**) LM-tumour cell migration toward the biglycan protein at 10 μg/mL in the presence of an anti-TLR2 or anti-TLR4 antibody (10 μg/mL) was evaluated by a migration assay (*P < 0.01, one-way ANOVA. Data are represented as mean ± SD, n = 8 fields). (**G**) LM-tumour cell migration toward monolayers of TECs with or without biglycan knockdown (*P < 0.01 versus siCtrl, two-sided Student’s t-test. Data are mean ± SD, n = 6 fields). (**H**) LM-tumour cell migration toward the biglycan protein in the presence of 10 μM of the NF-κB inhibitor, BAY11-7082, was evaluated by a migration assay (*P < 0.01, one-way ANOVA. Data are represented as mean ± SD, n = 4 fields). (**I**) LM-tumour cells were preincubated with BAY11-7082 or anti-TLR2 and/or TLR4 antibodies. After stimulation of biglycan, cells were lysed and the levels of phospho-NF-κB were determined by western blotting. (**J**) LM-tumour cell migration toward biglycan in the presence of 10 μM of the MEK inhibitor, U0126, was evaluated by a migration assay (*P < 0.01, one-way ANOVA. Data are represented as mean ± SD, n = 4 fields). (**K**) LM-tumour cells were preincubated with U0126 or anti-TLR2 and/or TLR4 antibodies. After stimulation of biglycan, cells were lysed and the levels of phosphor-ERK1/2 were determined by western blotting.

To determine whether enhanced tumour metastasis by HM-TECs was due to biglycan, biglycan expression in HM-TECs was stably knocked down (Supplementary Fig. S3D,E). LM-tumour cells were then subcutaneously implanted with either control- or biglycan knockdown-HM-TECs (Fig. 3A). We collected plasma samples from mice and the plasma biglycan levels were analysed. Co-implantation of control HM-TECs with LM-tumours resulted in increased plasma biglycan levels (Fig. 3B). In contrast, when shBiglycan HM-TECs were co-implanted, the plasma biglycan levels were reduced (Fig. 3B and Supplementary Fig. S3F). These results indicate that co-implanted HM-TECs are a source of increased biglycan *in vivo*. In mice bearing tumours co-implanted with shBiglycan HM-TECs, the numbers of CTCs and the occurrence of lung metastasis were dramatically decreased from those in the control group (Fig. 3C,D and Supplementary Fig. S3G). These results support our aforementioned results that HM-TEC-derived biglycan induces LM-tumour cells to metastasize. Moreover, the MVD of tumours co-implanted with shBiglycan HM-TECs decreased (Supplementary Fig. S3H), which suggests that biglycan from the ECs induced tumour angiogenesis.

### Biglycan enhances tumour cell migration through the activation of NF-κB and ERK signalling via TLRs

Tumour cells expressed the biglycan receptors Toll-like receptor (TLR) 2 and TLR4^25^,^26^ (Fig. 3E). Biglycan protein enhanced tumour cell migration (Fig. 3F) and this was inhibited by neutralizing the biglycan receptors using an anti-TLR2 or anti-TLR4 antibody (Fig. 3F). These results further confirmed that tumour cells are attracted by biglycan via TLR2 and TLR4. Both TLRs seems to be involved in tumour cell migration. Since there were no additive effects by inhibition of both TLR2 and TLR4, it was suggested that one of these TLRs may be sufficient for tumour cells to utilize biglycan. When biglycan was knocked down in HM-TECs, tumour cell migration towards HM-TECs was significantly decreased (Fig. 3G), thereby establishing the requirement for TEC-derived biglycan for tumour cell migration. In contrast, tumour cell adhesion to a monolayer of biglycan-knockdown HM-TECs did not change (Supplementary Fig. S3I).

To clarify the potential intracellular signalling cascade by which biglycan stimulates tumour cell migration, we investigated the effects of biglycan on the activation of NF-κB and ERK 1 and 2, known downstream biglycan signalling pathways^25^,^26^. Tumour cell migration toward biglycan was inhibited by the NF-κB inhibitor BAY11-7082 (Fig. 3H). Biglycan-treated tumour cells showed significant increases in NF-κB activity, whereas this induction was attenuated by pretreatment with TLR2 and/or TLR4 inhibitors (Fig. 3I), suggesting that biglycan-induced tumour cell migration was mediated by NF-κB activation through TLRs. Similarly, pretreatment of tumour cells with U0126, a specific inhibitor of mitogen-activated protein kinase (MEK) 1 and 2, decreased the number of tumour cells migrating toward biglycan (Fig. 3J). The enhanced activation of ERK1/2 in biglycan-stimulated tumour cells was abolished by preincubation with U0126 or TLR2 and/or TLR4 inhibitors (Fig. 3K). Taken together, these data suggest that the NF-κB and ERK pathways are involved in enhanced tumour cell migration by biglycan.

### Tumour blood vessels of patients with cancer express biglycan

**Figure 4:**
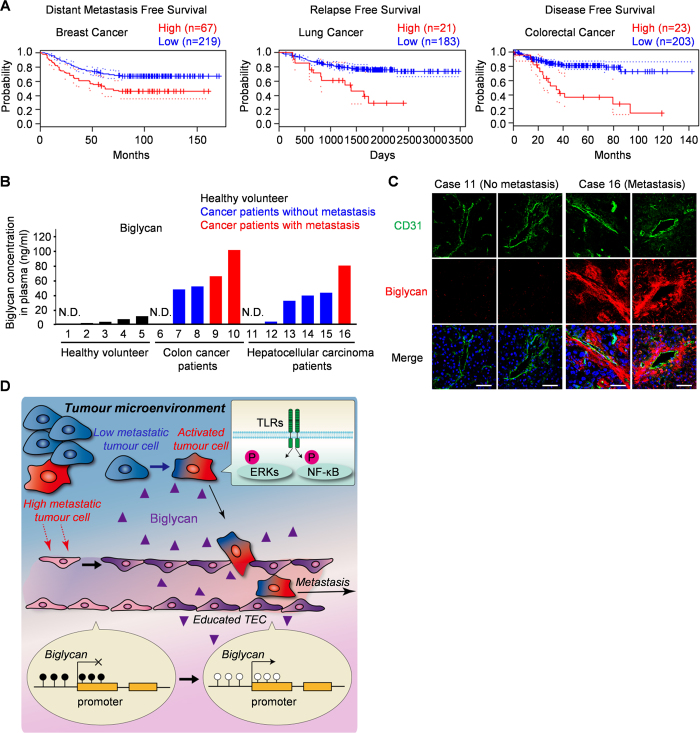
Tumour blood vessels of patients with cancer express biglycan. (**A**) Relationships between biglycan expression and the prognosis of patients with indicated cancer were investigated using the PrognoScan database (see also Table 1). Survival curves for high (red) and low (blue) expression groups divided at the optimal cutpoint are plotted. (**B**) Plasma biglycan levels were determined using ELISA for healthy volunteers (black columns), patients without metastatic cancer (blue columns) and patients with metastatic cancer (red columns). N.D., not detectable. (**C**) Representative tumour tissues were fixed, sectioned and stained with the anti-CD31 antibody (green) and the anti-biglycan antibody (red). Scale bar = 50 μm. See also Supplementary Fig. S3J. (**D**) Educated TECs affected by the tumour microenvironment of highly metastatic tumour cells provide a “gateway” for tumour cell metastasis.

The relationship between the gene expression and prognosis of patients with various cancers is available from the PrognoScan database^27^, a large collection of publicly available cancer microarray datasets with clinical backgrounds. As shown in Fig. 4A, we found that high biglycan expression was correlated with a poor prognosis in patients with breast cancer^28^, lung cancer^29^ and colorectal cancer^30^ (Table 1). Although the Cox p-value was not significant in lung cancer, the minimum p-value used to find the cutpoint showed this trend. Plasma biglycan levels in patients with cancer (Table 2) were higher than those in healthy volunteers (Fig. 4B). Among all samples tested, including those of patients with nonmetastatic cancer, the highest biglycan levels were found in patients with metastases (Fig. 4B, red columns). Thus, high biglycan expression may positively correlate with tumour progression. To determine the source of biglycan expression, we further assessed biglycan expression in individual cases. Strong biglycan expression was detected in tumour blood endothelial cells of a metastatic case (case number 16) but was barely detected in the tumour tissues of a nonmetastatic case (case number 11) (Fig. 4C and Supplementary Fig. S3J,K). Since biglycan is a secreted proteoglycan, biglycan was also stained in the surrounding areas of blood vessels in metastatic cases, which has been described in previous reports^31^. These data suggest that biglycan in TECs is also involved in tumour metastasis in patients with cancer.

## Discussion

In this study, we demonstrated that TECs isolated from high-metastatic tumours promoted lung metastasis of tumours that rarely metastasize on their own. Tumour cell intravasation was enhanced by TEC-derived biglycan and vice versa. Expression of biglycan was regulated by DNA demethylation, indicating a bidirectional interaction between tumour cells and TECs in the tumour microenvironment. In this regard, TECs are altered by the tumour microenvironment, which, in turn, creates a local milieu more favourable for tumour metastasis, including facilitated tumour intravasation (Fig. 4D).

Much remains to be elucidated regarding the determinants for tumour metastasis. A number of studies conducted during recent decades have challenged this paradigm by addressing tumour cell-autonomous mechanisms. Nevertheless, it remains difficult to predict metastasis. Tumour size and the histological grade, as evaluated by pathological examination, are not always linked with metastatic status. These observations encouraged us to examine the role of tumour stromal cells, as we focused on the properties of TECs in this study. Evidence gathered to date indicates that tumour stromal cells, such as cancer associated fibroblasts, promote tumour progression after co-implantation of these cells with tumour cells^32^,^33^. TECs, a type of tumour stromal cells, also exhibit aberrant behaviours^15^,^34^ and physically make contact with tumour cells during tumour metastasis (intravasation). Nevertheless, studies that evaluated the possibility that TECs affect tumour progression, particularly metastasis, have been limited. Since the TEC population is approximately only 2%^8^, we co-implanted TECs (2% of tumour cells) in our study. Our data demonstrate that even a small number of TECs display significant differences in metastatic potential and implicates TECs as strong environmental determinants of the metastatic potential of tumours.

Although we have previously shown that TECs from tumours with different metastatic potential had distinct properties^14^, it was not clear whether these phenotypic differences indeed contributed to specific aspects of tumour metastasis. Among previously reported TEC markers, we identified biglycan expressed in HM-TECs as a key molecule. In our experimental tumour model, both HM- and LM-tumour cells secreted vanishingly low levels of biglycan^1^, which established it as a suitable model for analysing the roles of biglycan derived from cells other than tumour cells. Our data revealed that TECs and other stromal cells, but not tumour cells, expressed biglycan in HM-tumours. Biglycan promoted tumour cell migration, and biglycan knockdown in HM-TECs inhibited tumour migration toward HM-TECs and decreased numbers of CTC and tumour metastasis. These results demonstrate that biglycan secreted from HM-TECs mediates tumour cell intravasation and subsequent metastasis. Given that the increased biglycan levels in plasma from mice with LM-tumour cells/HM-TECs was significantly reduced by biglycan knockdown in HM-TECs, HM-TECs are crucially involved in the induction of biglycan. We recently identified biglycan as a novel TEC marker contributing to the proangiogenic phenotype of TECs in an autocrine manner^23^. Biglycan is a component of the extracellular matrix, but is known to be released from activated macrophages in inflammatory and fibrotic tissues^25^,^35^,^36^,^37^. Biglycan has the capacity to bind transforming growth factor-β (TGF-β)^38^, tumour necrosis factor-α^39^ and bone morphogenetic protein-4^40^. Thus, stromal biglycan appears to modulate the biological activities of a variety of growth factors. Although it is still unknown whether biglycan secreted from TECs has the same function or not, the role of biglycan secreted from TECs is worth investigating in future studies. In addition, biglycan may also act as a chemoattractant for tumour cells, in addition to TECs^23^. Because it is a secreted protein and regulates inflammatory responses via its cognate TLR receptors in these situations^25^, we hypothesized that TEC-secreted biglycan attracted tumour cells in a paracrine manner. In support of this view, the tumour cells utilized in this study expressed TLR2 and TLR4, and neutralizing antibodies against these receptors indeed inhibited tumour cell migration toward biglycan, which suggested a common mechanism for biglycan to activate cell migration. NF-κB and ERK are involved in the intracellular signalling cascades of biglycan in macrophages through TLRs^25^,^26^. In our study, both NF-κB and ERK were involved in tumour cell migration. In addition, in HM-tumour tissues, increased numbers of macrophages from those in LM-tumour tissues were detected when analysed by F4/80 staining (data not shown). Macrophages are reported to be involved in tumour progression. In our study, macrophages may be mobilized by TEC-derived biglycan and this may contribute to tumour metastasis. The role of biglycan in macrophage mobilization and its consequence require further analysis.

HM-tumour CM treatment upregulated the expression of biglycan in LM-TECs and made LM-TECs attract more tumour cells, indicating a bidirectional interaction between tumour cells and TECs. It has been reported that TGF-β upregulates biglycan^41^,^42^,^43^. However, HM-tumour cells do not express TGF-β (data not shown), thus there may be another factor secreted from HM-tumours which causes upregulation of biglycan in TECs. The sustained high levels of biglycan expression in HM-TECs in culture encouraged us to analyse the epigenetic changes of a biglycan promoter in HM-TECs. Few reports have examined epigenetic differences in NECs and TECs^44^,^45^. In addition, there are few reports on the consequences of methylation of those genes in ECs. In the present study, we demonstrated for the first time differences in the DNA methylation patterns of biglycan between low- and high-metastatic tumour-derived ECs. These results suggest that the tumour microenvironment affects the epigenome in TECs. The mechanisms underlying the epigenetic alterations remain unknown, even in cancer cells. Similarly, the mechanisms underlying our findings are not yet clear, and further analysis is warranted. However, our findings provide a new prospect for understanding the mechanisms underlying abnormality in cancer stroma cells such as TECs. We have reported that biglycan expression in TECs is not limited to murine melanoma models but is also found in several other human solid tumours such as lung cancer and renal cancers^23^. In addition, high plasma concentrations of biglycan were observed in patients with colorectal and hepatocellular carcinomas with the highest concentrations in those with metastatic lesions. In breast, lung and colorectal cancers, high biglycan expression was correlated with poor prognosis by meta-analyses using PrognoScan. These results suggest a universal role for biglycan in facilitating metastasis across a wide range of tumours.

Although the detailed molecular mechanisms by which tumour cells acquire their metastatic potential remain to be determined, our results provide the first clear evidence for the importance of the different properties of TECs for determining tumour progression and/or malignancy. Tumour blood vessels act as a “gate” for metastasis (during intravasation of tumour cells), which is guarded by TECs as “gatekeepers.” TECs in highly metastatic tumours are thereby provided with a “key” molecule, biglycan, through DNA hypomethylation to allow tumour cells to break through this gate and proceed into the blood stream, which results in hematogenous metastasis (Fig. 4D). The present observations, together with unravelling certain remaining issues, may contribute to establishing accurate diagnostics or potent antimetastatic strategies that target the communications between tumour cells and endothelial cells.

## Materials and Methods

### Cell culture

Human malignant melanoma A375 cells (LM-tumour) were purchased from the American Type Culture Collection (ATCC). A375SM (super-metastatic) cells (HM-tumour) were kindly provided by Dr. Fidler (M.D. Anderson Cancer Centre, Houston, TX, USA). HM-tumour cells were established by *in vivo* selection of pulmonary metastatic lesions in nude mice and eventually display significantly high metastatic potentials, relative to those of the parental A375 cells^17^. These cells were cultured in Minimum Essential Medium (MEM, GIBCO) supplemented with 10% heat-inactivated fetal bovine serum (FBS). NIH3T3 and RAW264.7 cells were purchased from ATCC and DS Pharma Biomedical, respectively. These cells were cultured in Dulbecco’s Modified Eagle’s Medium (DMEM, Sigma) supplemented with 10% FBS. Cells were cultured at 37 °C in a humidified atmosphere containing 5% CO_2_. The absence of Mycoplasma pulmonis was checked by PCR at the ICLAS Monitoring Center.

### Mice

Six-week-old female nude mice (BALB/c AJcl-nu/nu, Clea, Japan) were housed under specific pathogen-free conditions. All procedures for animal care and experimentation adhered to institutional guidelines and were approved by the Ethical Committee for Experimental Animal Care of Hokkaido University.

### Isolation of TECs and NECs

TECs and NECs were isolated as previously described^14^,^19^, with modifications. Briefly, xenografts of HM- or LM-tumours in 10 nude mice and the dermis of non-tumour bearing mice were minced, after which ECs were sorted using an IMag cell separation system (BD Biosciences) with an anti-CD31 antibody. ECs were then maintained in EGM-2 MV (Lonza) containing 15% FBS. Diphtheria toxin (DT, 500 ng/mL, Calbiochem) was added to EC subcultures to eliminate any remaining human tumour cells that expressed heparin-binding EGF-like growth factor (HB-EGF), a DT receptor^15^. Isolated ECs were further purified using FITC-BS1-B4-lectin. CD31 (-) mouse stromal cells were also collected from HM-tumour xenografts and then added DT to eliminate tumour cells.

### Chemicals and antibodies

The following chemicals were purchased: fluorescein isothiocyanate (FITC)-Bandeiraea simplicifolia isolectin B4 (BS1-B4) (Vector Laboratories), Alexa Fluor 647-Griffonia simplicifolia 1 isolectin B4 (GS1-B4) (Invitrogen), Biglycan protein from bovine articular cartilage (Sigma-Aldrich), MEK inhibitor U0126 (CST) and NF-κB inhibitor BAY11-7082 (Calbiochem). The antibodies used are listed in Supplementary Table S1.

### Isolation of RNA, reverse transcription-PCR (RT-PCR) and quantitative PCR

Total RNA was isolated using an RNeasy Micro kit (Qiagen). cDNA was synthesized using ReverTra-Plus (Toyobo), as previously described^10^, and amplified by PCR. PCR products were visualized by ethidium bromide staining. Quantitative real-time RT-PCR was performed using SsoFast EvaGreen Supermix (Bio-Rad). Cycling conditions were set based on CFX Manager (Bio-Rad). mRNA expression levels were normalized to those of Gapdh and analysed using the delta-delta-Ct method. The primers used are listed in Supplementary Table S2.

### Flow cytometry analysis

TECs and NECs were incubated with an antibody against either BS1-B4, CD144, CD45, or CD11b, as previously described^14^, and analysed using a FACS Aria II (Becton Dickinson). Data were analysed using FlowJo software (Tree Star Inc.).

### Tube formation assay

ECs were seeded on Matrigel (BD Biosciences), as previously described^10^. Tube formation was observed using an inverted microscope (CKX41, Olympus).

### Cell migration assay

Tumour cell migration toward ECs was assessed using transwell chambers (Corning), as previously described^46^,^47^, with modifications. EC suspensions were placed into the lower compartment. After incubation for 6 h, the culture medium was replaced with fresh serum-free medium and the cells were incubated for an additional 24 h. Tumour cell suspensions maintained in serum-free medium for 24 h prior to assays were then placed in the upper compartment and incubated for 6 h. Tumour cell migration toward biglycan (chemoattractant) was analysed with a Boyden chamber, as previously described, with modifications^10^. Cells were pretreated with 10 μM of U0126 or BAY11-7082, if necessary, and then seeded in the upper chamber. The migrated cells were counted after 4 h. Non-migrating cells were removed with a cotton swab, followed by fixation with 10% formalin (Wako) and staining with Mayer’s hematoxylin (Wako). Cells that had migrated to the bottom surface were counted using a microscope.

### Adhesion assay

Tumour cells were pre-labelled with mouse anti-human HLA-ABC antibody, added to EC monolayers in a Lab-Tek 8-well Chamber Slide (Nalge Nunc International) and allowed to adhere for 30 min. After removing non-adherent cells, cells were fixed, stained with DAPI (Dojin) and counted under a fluorescence microscope.

### Transendothelial migration assay

Venus-expressing ECs were cultured on 35-mm glass-bottomed dishes (Matsunami Glass). RFP-expressing tumour cells were added to EC monolayers. After 2 h of co-culture, cells were fixed with 4% paraformaldehyde, covered with mounting medium (Vector Laboratories) and then examined using an Olympus FV1000 confocal microscope for a total of 20 fields. X-Z orthogonal reconstructions were made using FV10-ASW Viewer software. Tumour cells were scored according to the positional categories shown in Fig. 1A. The number of tumour cells at each stage was counted and expressed as a percentage of the total cell number.

### Plasmids and transfection

cDNA for Rluc, kindly provided by Y. Ohmiya (AIST), was amplified by PCR and cloned into pCR-Blunt II-TOPO (Invitrogen). The resulting PCR products were subcloned into the XhoI and NotI sites of pCAGGS-Venus^48^ and the DNA fragment encoding for Venus and Rluc was then subcloned into the EcoRI and NotI sites of pCS II-CMV-MCS (from H. Miyoshi, RIKEN). ptdTomato-C1 was purchased from Clontech, digested with AgeI and EcoRI and inserted into the AgeI-EcoRI sites of pCSII-CMV-MCS to generate pCSII-CMV-tdTomato. The following oligonucleotides were annealed, digested with BglII and XbaI and inserted into the BglII-Xbal sites of the Gateway entry vector pENTR4-H1 (Invitrogen): 5′-GATCTCCgaacatagccagatgaagaTTCAAGAGAtcttcatctggctatgttcTTTTTGGAAT-3′, 5′-CTAGATTCCAAAAAgaacatagccagatgaagaTCTCTTGAAtcttcatctggctatgttcGGA-3′ for sh-Biglycan; 5′-GATCTCCgttcactacctgtcaatccTTCAAGAGAggattgacaggtagtgaacTTTTTGGAAT-3′, 5′-“CTAGATTCCAAAAAgttcactacctgtcaatccTCTCTTGAAggattgacaggtagtgaacGGA”-3′ for sh-Biglycan#2. The sequences against GFP were used as a negative control. These entry clones were then transferred into attR sites in the lentiviral vector for RNA interference CS-RfA-CMV-mRFP1 (from H. Miyoshi) using LR Clonase (Invitrogen). The self-inactivating lentiviral vectors together with the packaging vector pCAG-HIVgp and the VSV-G- and REV-expressing construct pCMV-VSV-G-RSV-REV (from H. Miyoshi) were introduced into 293 T cells using FuGene HD (Promega), according to the manufacturer’s recommendations. Lentivirus-mediated gene transfer was performed, as previously described^49^.

### *In vivo* tumour metastasis model

A375 cells (1 × 10^6^) and ECs (2 × 10^4^) were subcutaneously implanted in the right flanks of nude mice. After 29 days, blood was collected from anesthetized mice by cardiac puncture. Circulating tumour cells were analysed using a FACS Aria II. Data were analysed using FlowJo software. Lungs were subjected to *ex vivo* bioluminescence imaging using IVIS Spectrum (Caliper Life Science). Tumour vessels were imaged using an Olympus SZX12 fluorescence stereomicroscope equipped with a RFP filter set (Olympus). DP20 (Olympus) and MicroMax 1300YHS (Princeton Instruments) under the control of MetaMorph software (Universal Imaging) were used to acquire bright-field (exposure time of 20 ms) and fluorescent images (1.0 s), respectively.

### Immunohistochemistry

Frozen sections of mouse tumour tissues were prepared, as previously described^14^, and double-stained using anti-CD31 and anti-biglycan antibodies followed by counterstaining with DAPI. Sample images were acquired using an IX71 microscope or an FV1000 confocal microscope (Olympus). The acquired images including line profiles were processed using Fluoview FV10-ASM Viewer software (Olympus). Microvessel density (MVD) of each CD31-stained tumour was determined, as previously described.

### Biglycan knockdown by siRNA

*Biglycan* siRNA (5′-AAACCCUUCUGCUCAAAGGGCAAGG-3′) was introduced into cells using Lipofectamine RNAiMAX Transfection Reagent (Invitrogen, Carlsbad, CA, USA). A non-targeting control siRNA (Invitrogen, Carlsbad, CA, USA) was used as a negative control.

### Western blotting

ECs were grown to subconfluence and culture medium was replaced with fresh EGM-2 MV. After 18–24 h, CM was collected and passed through a 0.22-μm filter (Merck Millipore) and subsequently concentrated approximately 120-fold using Amicon Ultra-15 30 K centrifugal filter units and Amicon Ultra 0.5-mL 30 K centrifugal filters (Merck Millipore). LM-tumour cells were preincubated with anti-TLR2 and/or TLR4 antibodies (Biolegend) for 90 min, or with U0126 or BAY11-7082 for 60 min. Cells were lysed after stimulation of biglycan for 30 min (ERK) or 60 min (NF-κB). Equal amounts of total protein were separated by sodium dodecyl sulphate polyacrylamide gel electrophoresis (SDS-PAGE) and transferred to polyvinylidene difluoride (PDVF) membranes. Equal loading and transfer were confirmed by MemCode Reversible Protein Stain Kit (Thermo Scientific) for detection of biglycan in the CM of ECs. Western blotting was performed using antibodies listed in Supplementary Table S1 and an HRP-conjugated secondary antibody, as previously described^10^,^12^.

### Enzyme-linked immunosorbent assay (ELISA)

Plasma biglycan concentrations were determined using a sandwich enzyme immunoassay for mouse or human biglycan (Uscn, Life Science Inc.).

### Bioinformatics

The correlation between biglycan expression levels and the prognosis of patients was obtained from the PrognoScan database^27^, which is a large collection of publicly available cancer microarray datasets with clinical backgrounds. In brief, patients were divided based on biglycan expression levels into high- and low- expression groups. Differences in risk between any two groups were estimated by the log-rank test and shown in Table 1.

### Human blood and tissue samples

Blood was collected from patients who were clinically diagnosed with colon cancer or hepatocellular carcinoma (Table 2) and from healthy volunteers. Tumour tissues were surgically resected. All protocols were approved by the Institutional Ethics Committee of Hokkaido University and written informed consent was obtained from each patient before surgery. Final diagnosis of the cases was confirmed by pathological examination of formalin-fixed surgical specimens. All the methods involving humans were carried out in accordance with the relevant guidelines, including any relevant details.

### MSP

Genomic DNA was extracted using NucleoSpin Tissue (Macherey-Nagel). The extracted DNA was bisulfite-treated using an EpiTect Bisulfite kit (Qiagen) and amplified with methylation- or unmethylation-specific primers designed with MethPrimer software. The primers are listed in Supplementary Table S3. PCR reactions were performed using an EpiScope MSP kit (Takara Bio). Mouse high-methylated genomic DNA and low-methylated genomic DNA (EpigenDx) were used as positive and negative controls, respectively.

### Bisulfite DNA sequencing

Genomic DNA samples were treated with bisulfite using a MethylEasy Xceed Rapid DNA Bisulphite Modification kit (Takara Bio) and then used as a template for PCR amplification of the region of interest. The primer pairs are listed in Supplementary Table S3. PCR products were purified and cloned into pUC118 Hinc II/BAP vectors (Takara Bio). Individual clones were sequenced and aligned with a reference sequence. Methylation pattern figures were generated using QUMA software (http://quma.cdb.riken.jp/top/quma_main_j.html).

### 5-aza-dC treatment

The demethylating agent 5-aza-2′-deoxycytidine (5-aza-dC) (Sigma) was added to the culture medium and incubated for 3 days.

### Statistics

All data, unless otherwise specified, are expressed as the mean ± standard deviation (SD) of three independent experiments performed in triplicate and subjected to one-way ANOVA, followed by a Tukey–Kramer multiple comparison test. A two-sided Student’s t-test was used for comparison between two groups.

## Additional Information

**How to cite this article**: Maishi, N. *et al*. Tumour endothelial cells in high metastatic tumours promote metastasis via epigenetic dysregulation of biglycan. *Sci. Rep.* **6**, 28039; doi: 10.1038/srep28039 (2016).

## Supplementary Material

Supplementary Information

## Figures and Tables

**Table 1:** Relationships between biglycan expression and prognosis of patients with breast cancer, lung cancer and colorectal cancer were investigated using the PrognoScan database.

DATASET	GSE2034	GSE31210	GSE14333
CANCER TYPE	Breast cancer	Lung cancer	Colorectal cancer
N	286	204	226
ENDPOINT	Distant Metastasis Free Survival	Relapse Free Survival	Disease Free Survival
COHORT	Rotterdam (1980-1995)	NCCRI	Melbourne
ARRAY TYPE	HG-U133A	HG-U133_Plus_2	HG-U133_Plus_2
PROBE ID	201261_x_at	213905_x_at	201262_x_at
CONTRIBUTOR	Wang	Okayama	Jorissen
CUTPOINT	0.77	0.9	0.9
MINIMUM *P*-VALUE	0.000553	0.000282	0.000001
CORRECTED *P*-VALUE	0.015452	0.008652	0.000066
IN (HR_high_/HR_low_)	0.69	1.13	1.39
COX *P*-VALUE	0.012793	0.611805	0.005755
In (HR)	0.33	0.16	0.27
HR [95% CI]	1.4 [1.07–1.81]	1.18 [0.63–2.20]	1.31 [1.08–1.59]

**Table 2:** Clinical backgrounds of colon cancer and hepatocellular carcinoma specimens.

Colon cancer										
Case no.	Age	Sex	T	N	M	v	Stage			
6	65	M	3	1	0	−	IIIa			
7	71	M	3	0	0	−	II			
8	67	F	3	2	0	−	IIIb			
9	47	F	4a	0	1 (liver)	+	IV			
10	69	M	3	0	1 (liver)	+	IV			
**Hepatocellular carcinoma**										
**Case no.**	**Age**	**Sex**	**T**	**N**	**M**	**im**	**vp**	**vv**	**va**	**Stage**
11	80	M	2	0	0	−	−	−	−	II
12	62	M	2	0	0	−	−	−	−	II
13	74	M	2	0	0	−	−	−	−	II
14	77	M	2	0	0	−	−	−	−	II
15	61	M	2	0	0	−	−	−	−	II
16	63	M	4	0	0	+	+	−	−	IIIc
