# Supplementary material for: Tumour endothelial cells in high metastatic tumours promote metastasis via epigenetic dysregulation of biglycan
Source: Sci Rep. 2016 Jun 13;6:28039. doi: 10.1038/srep28039 (PMC4904795; doi:10.1038/srep28039)
Supplement: Supplementary Information [file srep28039-s1.pdf]

Tumour endothelial cells in high metastatic tumours promote metastasis via epigenetic dysregulation of biglycan

Nako Maishi[1,2], Yusuke Ohba[3], Kosuke Akiyama[1,2], Noritaka Ohga[2], Jun-ichi Hamada[4], Hiroko Nagao-Kitamoto[2], Mohammad Towfik Alam[1,2], Kazuyuki Yamamoto[2], Taisuke Kawamoto[2], Nobuo Inoue[5], Akinobu Taketomi[6], Masanobu Shindoh[7], Yasuhiro Hida[8] and Kyoko Hida[1,2*]

Supplementary Table S1. List of primary antibodies employed in flow cytometry, immunohistochemistry, immunocytochemistry and immunoblotting.

	Antibodies	Application/Dilution	Catalogue No	Clone	Supplier
1	anti-mouse CD31	FC 1:400, IHC 1:400	553370	MEC 13.3	BD Pharmingen
2	anti-human vimentin	IHC 1:1000	HPA001762	A35145	SIGMA
3	Alexa Fluor 647-anti-mouse CD31	IHC 1:400	102416	390	Biolegend
4	anti-mouse CD144	FC 1:400	555289	11D4.1	BD Pharmingen
5	APC-anti-mouse CD45	FC 1:400	103112	30-F11	Biolegend
6	APC-anti-mouse CD11b	FC 1:400	17-0112-81	M1/70	eBioscience
7	FITC-anti-human HLA-ABC	ICC 1:100	555552	G46-2.6	BD Pharmingen
8	anti-biglycan	IB 1:2000	ab58562	polyclonal	Abcam
9	anti-biglycan	IHC 1:1000	ab49701	polyclonal	Abcam
10	anti-β-actin	IB 1:1000	#4970	13E5	Cell Signaling
11	anti-phospho-NFκB p65 (Ser536)	IB 1:1000	#3033	polyclonal	CST
12	anti-NFκB p65	IB 1:1000	#8242	polyclonal	CST
13	anti-phospho-ERK1/2	IB 1:2000	#4370	polyclonal	CST
14	anti-ERK1/2	IB 1:1000	#9102	polyclonal	CST

Abbreviations

FC: flow cytometry, IHC: immunohistochemistry, ICC: immunocytochemistry, IB: immunoblotting

Supplementary Table S2. List of primers for PCR analysis.

NAME OF GENES	SEQUENCE
Mouse Gapdh	forward 5'-TCTGACGTGCCGCCTGGAG-3'
	reverse 5'-TCGCAGGAGACAACCTGGTC-3'
Mouse Cd31	forward 5'-TGCTCTCGAAGCCCAGTATT-3'
	reverse 5'-ATGGGTGCAGTTCCATTTTC-3'
Mouse Cd105	forward 5'-CTTCCAAGGACAGCCAAGAG-3'
	reverse 5'-GGGTCATCCAGTGCTGCTAT-3'
Mouse Vegfr1	forward 5'-GAGGAGGATGAGGGTGTCTATAGGT-3'
	reverse 5'-GTGATCAGCTCCAGGTTTGACTT-3'
Mouse Vegfr2	forward 5'-GCCCTGCTGTGGTCTCACTAC-3'
	reverse 5'-CAAAGCATTGCCCATTCGAT-3'
Mouse Biglycan	forward 5'-AACTCACTGCCCCACCACAGCTTC-3'
	reverse 5'-GCGGTGGCAGTGTGCTCTATCCATC-3'
Human GAPDH	forward 5'-ACAGTCAGCCGCATCTTCTT-3'
	reverse 5'-GCCCAATACGACCAAATCC-3'
Human HB-EGF	forward 5'-GCGGGCTGAGTGAGCAAGACAAGAC-3'
	reverse 5'-AGGCACCAGTCACTTTCGAAGCGG-3'
Human TLR2	forward 5'-CCTCCAATCAGGCTTCTCTG-3'
	reverse 5'-TCCTGTTGTTGGACAGGTCA-3'
Human TLR4	forward 5'-AGTCCATCGTTTGGTTCTGG-3'
	reverse 5'-CAATGGTCAAATTGCACAGG-3'

Supplementary Table S3. Primer pairs used for MSP and Bisulfite sequencing.

Primer sets used for MSP analysis
M-forward 5'-GTTTTCGGTTGGTTTTGAC-3'
M-reverse 5'-CAATTCCCGACGTAAACA-3'
U-forward 5'-GTTGTTTTTGGTTGGTTTTGAT-3'
U-reverse 5'-CAATTCCCAACATAAACAAAC-3'
**Primer sets used for Bisulfite sequencing**
Forward 5'-TAAATTATGTTTGAGGTAGGGG-3'
Reverse 5'-AAAACAACAAAATTACCAACATC-3'

Supplementary Figure S1

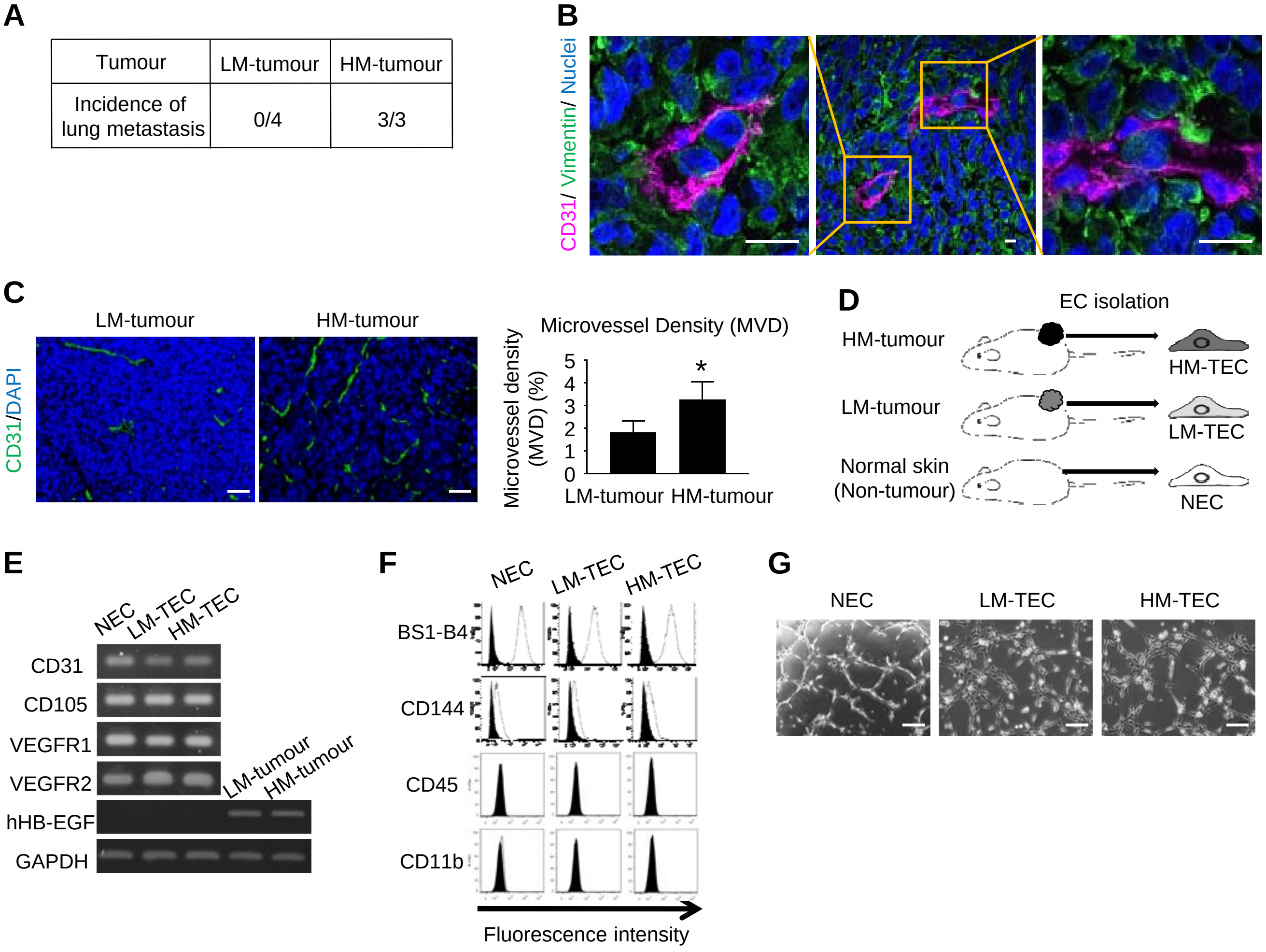

Tumour	LM-tumour	HM-tumour
Incidence of lung metastasis	0/4	3/3

Supplementary Figure S1. Characterization of tumour cells and isolated ECs. (A) Lung metastasis occurrence was determined on day 28 after LM-tumours and HM-tumours were subcutaneously xenografted into mice. (B) Sections prepared from HM-tumour xenografts were stained with anti-CD31 and anti-vimentin antibodies and counterstained with DAPI. Higher magnification images are also shown. Scale bar = 10 µm. Note that tumour cells were detected in intra-tumour blood vessel areas. (C) Angiogenic activity was compared between HM-tumours and LM-tumours by immunohistochemical analysis for microvessel density (MVD) using an anti-CD31 antibody. Representative data are shown in left panels. Scale bar = 100 µm. (*$P < 0.01$ versus LM-tumour, two-sided Student's t-test. Data are mean±SD, n=6 fields). (D) TECs were isolated from HM- and LM-tumour xenografts grown in nude mice. NECs were also isolated from the dermis of tumour-free nude mice and used as a normal control. (E) Cd31, Cd105, Vegfr1, Vegfr2, and hHB-EGF mRNA levels in TECs and NECs were determined by RT-PCR. LM-tumours and HM-tumours were used as positive controls for hHB-EGF. (F) Each EC type indicated at the top was incubated with BS1-B4 and antibodies against CD144, CD45 and CD11b and subjected to flow cytometry. Note that BS1-B4 binding, CD144 expression and a lack of CD45 and CD11b expression (white areas) indicated the high purity of isolated TECs and NECs. Data for cells treated with corresponding isotype control antibodies are shown as black areas. (G) Tube formation assay for each EC type on Matrigel. Scale bar = 100 µm.

Supplementary Figure S2

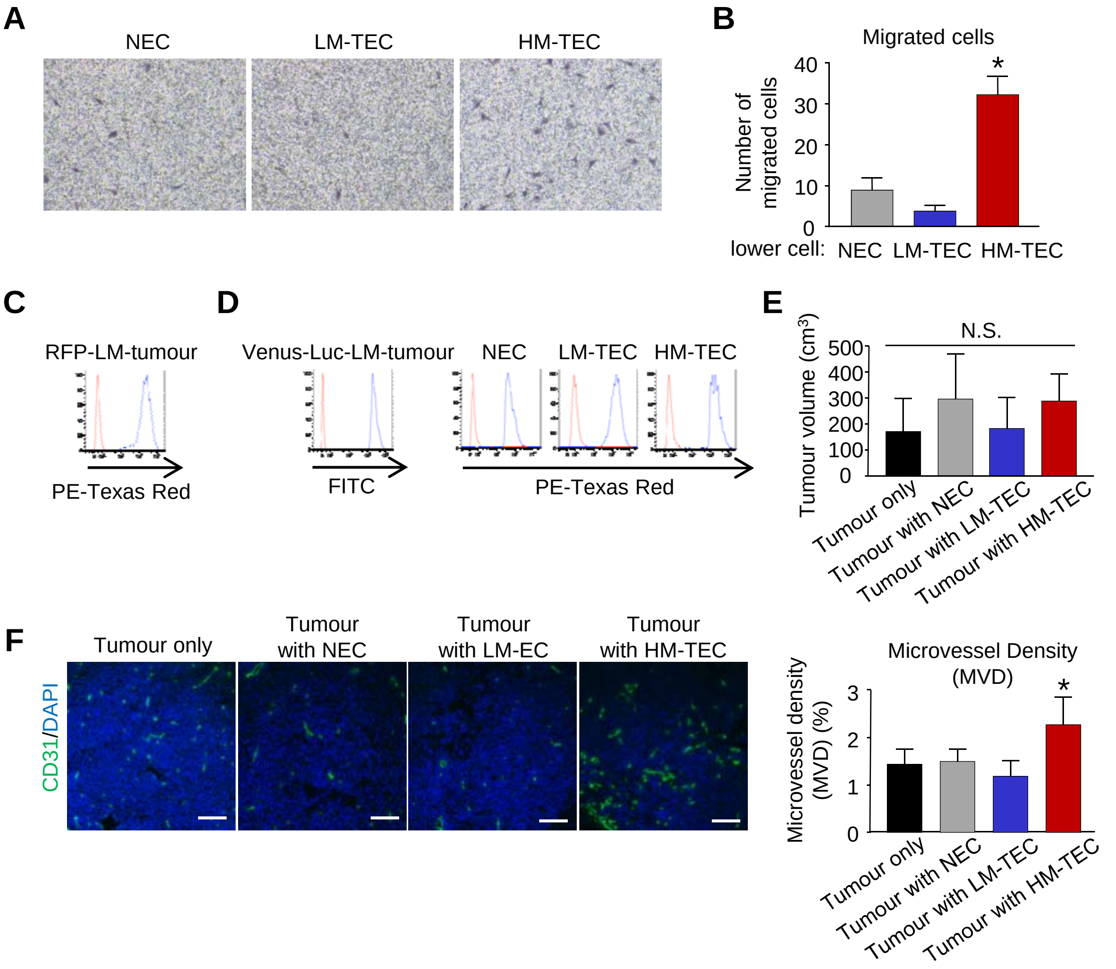

Supplementary Figure S2. The effect of each EC on tumours. (A and B) HM-tumour cells that migrated to the underside of a membrane were photographed (A) and counted (B). (*P < 0.01 versus LM-TECs and NECs, one-way ANOVA. Data are represented as mean ± SD., n = 6 fields). (C and D) Flow cytometry analysis of tumour cells and ECs, which were transfected with lentiviral vectors encoding for fluorescent proteins to distinguish one from the other (blue lines). Non-transfected cells (red lines) were used as control. (C) RFP-LM-tumour cells, (D) Venus-Luc-LM-tumour cells and tdTomato-ECs. (E) LM-tumour cells were xenografted with or without ECs as indicated. Tumour size was measured by surface examination at 29 days post-implantation. Note that there were no significant differences in tumour size among these groups (one-way ANOVA, N.S., not significant. Data are mean±SD, n = 4 or 5). (F) Microvessel density (MVD) of indicated tumour group was analysed using an anti-CD31 antibody (*P < 0.01 versus groups Tumour only, Tumour with NEC and Tumour with LM-TEC, one-way ANOVA. Data are mean±SD, n = 8 fields). Representative data are shown in left panels. Scale bar = 200 μm.

Supplementary Figure S3

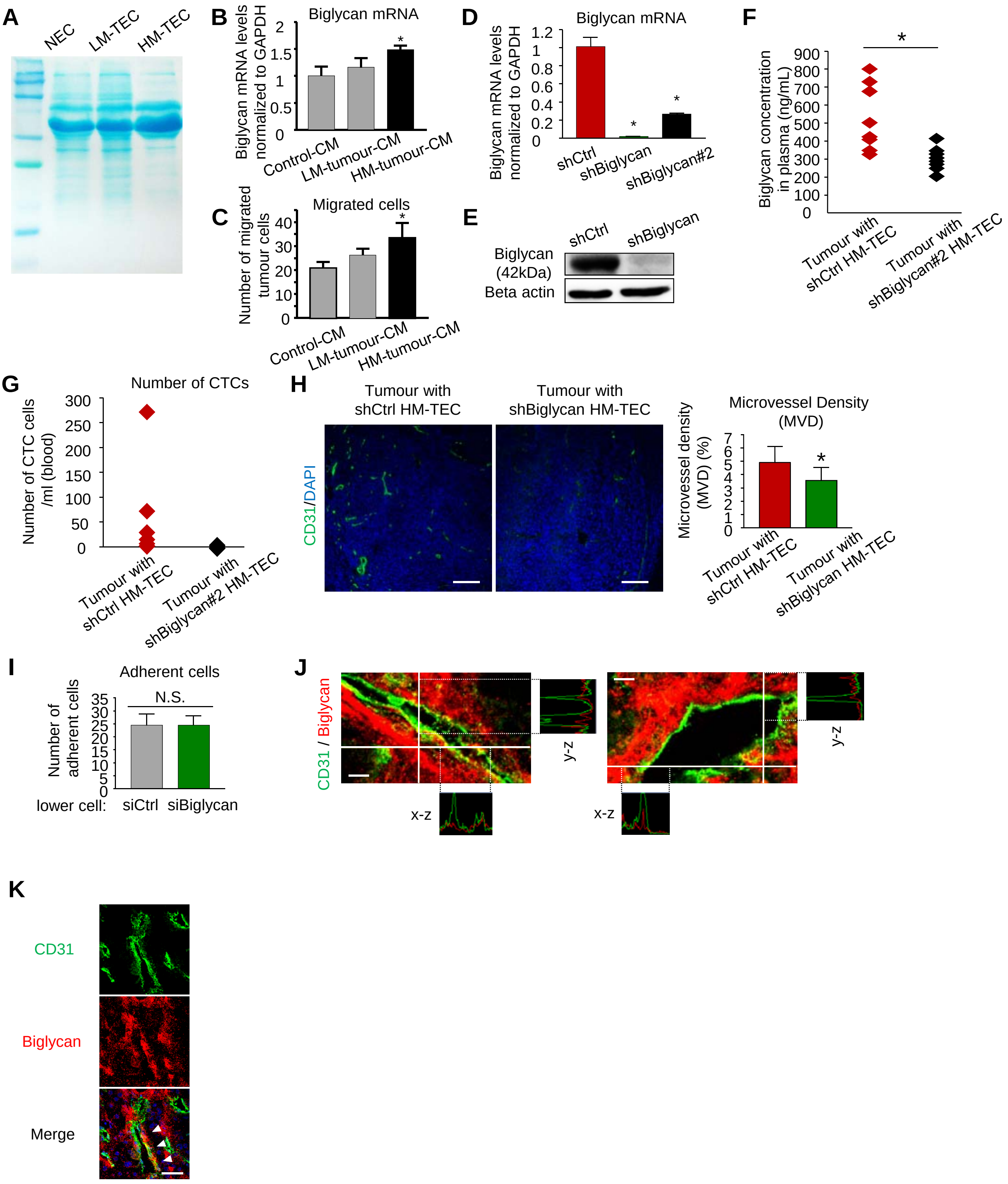

Supplementary Figure S3. HM-TEC-derived biglycan induces tumour cell intravasation and metastases. (A) Representative membrane stained for total protein to demonstrate equal loading. (B and C) LM-TECs were treated either with HM-tumour-CM, LM-tumour-CM or CM from LM-TECs as a control (Control-CM) for 2 days. (B) Biglycan mRNA expression levels were determined by real-time PCR (**P* < 0.01 versus Control-CM, one-way ANOVA. Data are mean±s.d., n = 4 real-time RT-PCR runs). (C) The numbers of migrating LM-tumour cells were determined using a transwell migration assay (**P* < 0.01 versus Control-CM, one-way ANOVA. Data are mean±s.d., n = 10 fields). (D and E) Biglycan expression in HM-TECs was stably knocked-down using lentiviral vector-mediated induction of shRNA. Biglycan knockdown in HM-TECs was confirmed at both the mRNA (D) and protein (E) levels (*P < 0.01 versus shCtrl, one-way ANOVA. Data are represented as mean ± SD, n = 4 real-time RT-PCR runs). (F) Plasma biglycan levels were determined by ELISA for each mouse group (*P < 0.01, two-sided Student’s t-test. n = 8). (G) The number of Venus-positive circulating tumour cells in Tumour with shCtrl HM-TEC and Tumour with shBiglycan#2 HM-TEC was analysed by flow cytometry (n = 8). (H) Indicated tumour tissues were fixed, sectioned and stained with an anti-CD31 antibody. Representative data are shown in left panels. Scale bar = 200 µm. MVD of Tumours co-implanted with HM-TECs transfected with shBiglycan or those with shCtrl were shown in right panel (*P < 0.01 versus Tumour with shCtrl HM-TEC, two-sided Student’s t-test. Data are mean±SD, n = 20 fields). (I) LM-tumour cell adhesion to monolayers of HM-TECs with or without biglycan knockdown was determined by an adhesion assay (one-way ANOVA, N.S., not significant. Data are mean±SD, n = 6). (J) Merged images for CD31 (green) and biglycan (red). Co-localization of CD31 and biglycan were also indicated by confocal line profile in lower and right panels. Scale bar = 10 µm. (K) Representative tumour tissues of Case 16 were fixed, sectioned, and stained with the anti-CD31 antibody (green) and the anti-biglycan antibody (red). Arrowhead indicates CD31 and biglycan co-localization. Scale bar = 50 µm. See also Figure 4C.
